# Loss & Gain

**Published:** 1879-07

**Authors:** 


*Loss and Gain.*—The immense advance in the profession of dentistry in the adaptation of machine-work, the fertility of inventive genius in producing instruments and appliances to facilitate the performance of operations upon the teeth, and the ready access by almost all to the sources of supply—the dental depots—have made changes which are working in more ways than one. As seen by the average practitioner, and as estimated by most of those whose practice "drives" them, the introduction of the dental engine, of the automatic plugger, the electric mallet, &c., are helps such as the busy man feels have grown out of the demands of the times, and out of the necessities of his calling. He feels that he could not do without them, wonders how he ever did, and smiles in quiet contempt of the "old fogy" who is unprovided with the "recent inventions."

But, if these things are real gains, if the labor-saving machinery and appliances for the rapid execution of work are positive advantages, and the dentist as well as the patient is advantaged by them, there is a loss to those of the profession who would under favorable conditions develop in prominence, so far counterbalancing the gains as to set one to seriously thinking if the gain is a satisfactory one, and compensating in effect.

The words "immense advance" used in the first line of this article are worth pondering over. They are used in this place in the popular sense. That the appliances and "engines" put into the hands of the young dentist are of ingenious construction and almost faultless mechanism no one can doubt who inspects them. But while they are useful to the average operator, *he who wishes to rise to a higher place, and develop the best of what is in him, will surely find these things a hindrance.*

Handicraft and deftness always fall off and are injured by machine-work. The machine will produce fairly well, but the best, and consequently rare, productions are the result of *genius guiding the hand.*

These thoughts are commended to the young of the profession, who are ambitious to aspire to burring engines and mallets. What the young want above all is hand-culture. No machine will take its place; and as well had the pupil at the piano undertake the fantasias of the master, in the hope of solid development as the student of dentistry hope to train the hand to that perfection of manipulation and delicacy of touch requisite to the production of the best work, by the use of machines of any sort, save such as resemble in handling the tools of the engraver. If we are gaining in facility, we are losing in deftness; and it is time that the teachers were pondering the question, not how fast can we introduce engines, &c., into the infirmaries and clinics, but how fast can we check their use. The future destiny of a manipulative profession like ours *lies in the ideal production of its masters.* These are few. Count all you know in dentistry on your fingers. Will you use all on one hand? The tendency of machines is to level, and will make average, but not first class operators; and you cannot profitably imitate your neighbor unless he is higher than yourself.

And if any young man of this day who reads these lines, wishes to rise to the height attained by a select few, if his aspirations are to develop all the latent genius resident in him, let him earnestly consider the means by which those who are his ideals were developed; let him scrutinize the practice of those who are of mediocre standing: and as a rule he will find that the best men use their brains and fingers, the mediocre men the brains and fingers of others, represented in our day largely by machines. H.

